# 3-Dimensional Immunostaining and Automated Deep-Learning Based Analysis of Nerve Degeneration

**DOI:** 10.3390/ijms232314811

**Published:** 2022-11-26

**Authors:** Sienna S. Drake, Marc Charabati, Tristan Simas, Yu Kang T. Xu, Etienne J. P. Maes, Shan Shan Shi, Jack Antel, Alexandre Prat, Barbara Morquette, Alyson E. Fournier

**Affiliations:** 1 Department of Neurology/Neurosurgery, Montréal Neurological Institute, McGill University, Montreal, QC H3A 2B4, Canada; 2 Centre de Recherche du Centre Hospitalier de l’Universite de Montreal, University of Montreal, Montreal, QC H2X 0A9, Canada

**Keywords:** multiple sclerosis, axon degeneration, deep-learning, image segmentation, optic neuritis, neuroinflammation, 3D-immunostaining, iDISCO, optic nerve

## Abstract

Multiple sclerosis (MS) is an autoimmune and neurodegenerative disease driven by inflammation and demyelination in the brain, spinal cord, and optic nerve. Optic neuritis, characterized by inflammation and demyelination of the optic nerve, is a symptom in many patients with MS. The optic nerve is the highway for visual information transmitted from the retina to the brain. It contains axons from the retinal ganglion cells (RGCs) that reside in the retina, myelin forming oligodendrocytes and resident microglia and astrocytes. Inflammation, demyelination, and axonal degeneration are also present in the optic nerve of mice subjected to experimental autoimmune encephalomyelitis (EAE), a preclinical mouse model of MS. Monitoring the optic nerve in EAE is a useful strategy to study the presentation and progression of pathology in the visual system; however, current approaches have relied on sectioning, staining and manual quantification. Further, information regarding the spatial load of lesions and inflammation is dependent on the area of sectioning. To better characterize cellular pathology in the EAE model, we employed a tissue clearing and 3D immunolabelling and imaging protocol to observe patterns of immune cell infiltration and activation throughout the optic nerve. Increased density of TOPRO staining for nuclei captured immune cell infiltration and Iba1 immunostaining was employed to monitor microglia and macrophages. Axonal degeneration was monitored by neurofilament immunolabelling to reveal axonal swellings throughout the optic nerve. In parallel, we developed a convolutional neural network with a UNet architecture (CNN-UNet) called BlebNet for automated identification and quantification of axonal swellings in whole mount optic nerves. Together this constitutes a toolkit for 3-dimensional immunostaining to monitor general optic nerve pathology and fast automated quantification of axonal defects that could also be adapted to monitor axonal degeneration and inflammation in other neurodegenerative disease models.

## 1. Introduction

The eye, oft referred to as ‘the window to the soul’, would be more aptly described as the window to the brain, as it is the most accessible component of the central nervous system (CNS). The eye houses the retina—a neural tissue composed of various cell types including retinal ganglion cells (RGCs) [1]. RGCs receive visual information from upstream neurons in the retina, and project their axons along the length of the optic nerve (ON) to the brain to deliver information to higher processing centers [1]. Simple and non-invasive techniques to monitor measures of retinal health including optical coherence tomography (OCT) can provide information about CNS health that are valuable to measure longitudinal outcomes in patients and animal models; however, higher resolution studies to study cellular pathology are still reliant on tissue fixation and analysis.

Multiple sclerosis (MS) is a chronic autoimmune disease of the CNS characterized by neuroinflammation and neurodegeneration [2]. In addition to various symptoms of neurological dysfunction linked to brain and spinal cord pathology, visual dysfunction is a common symptom in MS patients. Indeed, optic neuritis is diagnosed in up to 75% of MS patients over the course of their disease [1], and post-mortem assessment of ONs from MS patients reveal extensive ON pathology, including markers of extensive immune cell infiltration, reactive microgliosis and axonal degeneration, mirroring the pathological hallmarks of MS In CNS parenchyma [3]. Studying the eye is also useful to understand disease progression in MS [2]. Retinal nerve fibre layer (RNFL) thinning measured by OCT is associated with cognitive performance, quality of life metrics, and disability progression at 5 and 10 years [4,5,6]. Microperimetry, a non-invasive eye measurement, has been used to detect early sub-clinical retinal degeneration in MS patients [7], and MRI measurements of the ON in MS suggest widespread neuroaxonal degeneration associated with RNFL thinning [8]. Even asymptomatic ON lesions, where patients have optic nerve lesions with no history of optic neuritis, are associated with a thinner RNFL and decreased visual contrast sensitivity, and transorbital ultrasonography suggests a decrease in ON diameter in MS patients [9,10]. RNFL and ganglion cell layer degeneration is correlated with decreased brain volume in MS patients, and OCT measurements have been used to monitor effectiveness of therapies in preventing retinal atrophy [11,12,13,14]. OCT, MRI, level of disability, and visual evoked potentials are all correlated with decreases in low contrast visual sensitivity in MS patients [15].

Experimental autoimmune encephalomyelitis (EAE) is a preclinical MS disease model, and importantly, mice subjected to an EAE protocol demonstrate thinning of the RNFL, presence of ON lesions, and decreased visual acuity associated with the disease course, comparable to MS [16,17,18,19,20]. Histopathological studies have described extensive inflammation and immune cell infiltration in the retina and ON of EAE animals, demyelination of the ON, RGC death and axonal degeneration [17,21,22,23]. Therefore, the EAE mouse model is a useful tool to gain insight into potential molecular and pathological mechanisms underlying visual dysfunction in MS [24]. Assessing optic nerve pathology with traditional sectioning and staining techniques presents some challenges. Individual axon bundles cannot be easily followed since they may travel in and out of the plane of sectioning and subsequently axonal blebs cannot easily be differentiated from obliquely sectioned axons. Further, it is difficult to obtain sections that traverse the entire length of the ON resulting in spatial sampling bias. The orientation of the ON (ON head to chiasm) may also be lost during processing because of the symmetry of the optic nerve.

Immunolabeling-enabled three-dimensional imaging of solvent-cleared organs (iDISCO) is a tissue clearing technology that was developed to create optically transparent tissues enabling microscope imaging of tissues in three dimensions (3D) [25]. Here, we present an iDISCO protocol optimized for ON that maintains spatial orientation and enables 3D imaging of axonal tracts. In addition, we developed a computational neural network called BlebNet to quantify axonal defects quickly and accurately from confocal images of 3D neurofilament labelled ON. These methods provide a toolkit to monitor optic nerve pathology and quantify axonal degeneration in 3D that could be easily adapted to monitor axonal degeneration and inflammation in additional neurodegenerative disease models.

## 2. Results

### 2.1. Optimized iDISCO Protocol for Labelling Mouse Optic Nerve

**Figure 1:**
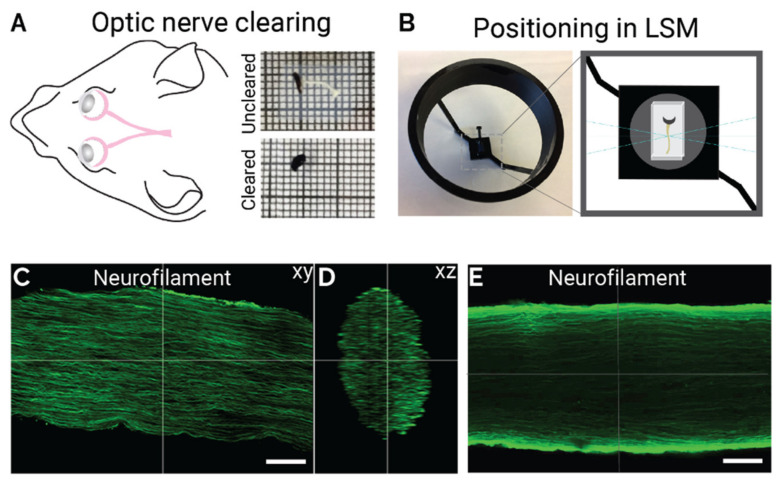
Optimized iDISCO for light sheet imaging of mouse optic nerve. (**A**) the optic nerve diagram is shown alongside the optic nerve before clearing (white in colour attached to the black globe of the eye) and after clearing (transparent, only globe is visible). (**B**) Positioning of the agar embedded optic nerve block in the light sheet microscope stage. (**C**–**E**) Representative max projection of optic nerves labelled with neurofilament, using either a single pre-conjugated antibody (**C**,**D**) or a separate primary and secondary antibody (**E**). Scale bar = 100 μm.

iDISCO is a technique that employs tissue clearing methods to make samples optically transparent for 3D imaging. Herein, a protocol for iDISCO was adapted for use with mouse optic nerve. Optic nerves were dissected with part of the globe attached to visualize the nerve and preserve its orientation in its transparent state following the clearing protocol (Figure 1A). Embedding the nerve in agarose after clearing enabled mounting in the light sheet microscope sample holder in a standardized manner and kept the nerves in the same orientation for imaging (Figure 1A,B). Two different immunohistochemistry protocols were tested: a one-step process using a pre-conjugated primary antibody, and a two-step process using a separate primary and secondary antibody. Using a single pre-conjugated antibody resulted in an even fluorescent signal throughout the optic nerve volume, whereas using a separate primary and secondary antibody resulted in brighter signal on the edges of the optic nerve volume suggesting uneven penetration of the antibody through the tissue (Figure 1C–E).

### 2.2. 3D Imaging of Immune Cell Infiltration and Inflammation in EAE

**Figure 2:**
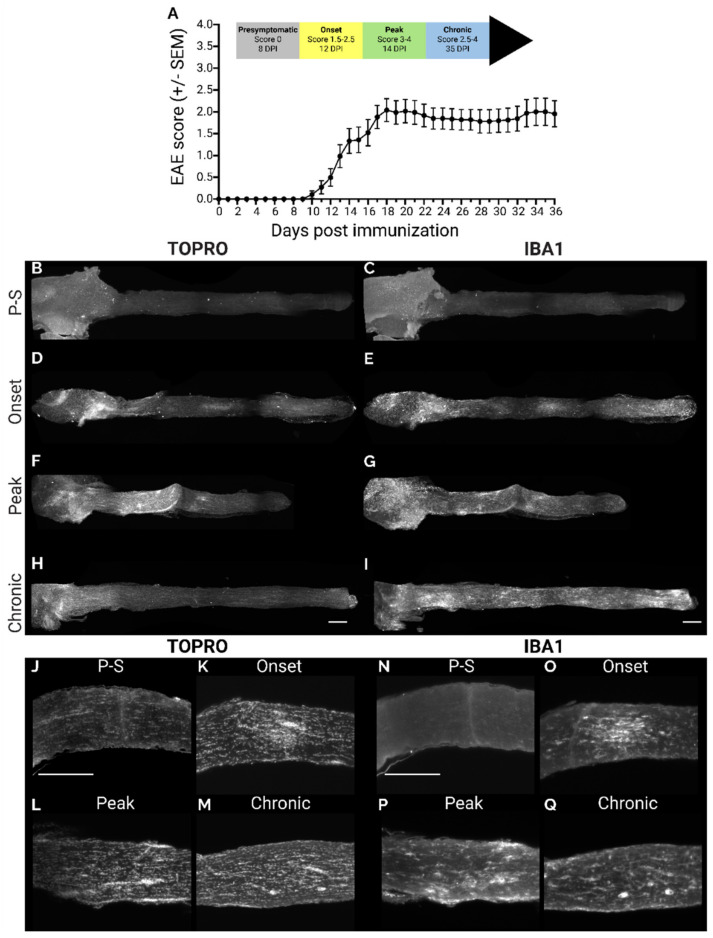
3D imaging of optic nerve inflammation in EAE. (**A**) EAE cohort score progression. (**B**–**I**) Representative max projection of straightened optic nerves labelled with Topro (**B**,**D**,**F**,**H**) or Iba1 (**C**,**E**,**G**,**I**) from pre-symptomatic; P-S (**B**,**C**), onset (**D**,**E**), peak (**F**,**G**), and chronic (**H**,**I**) stages of EAE. Single optical sections from Topro (**J**–**M**) or Iba1 (**N**–**Q**) labelled optic nerves in pre-symptomatic; P-S (**J**,**N**), onset (**K**,**O**), peak (**L**,**P**), and chronic (**M**,**Q**) stages of EAE. Scale bars = 500 μm.

Cleared whole optic nerves from EAE mice were immuno-stained and imaged in 3D at clinically defined timepoints: naïve (non-EAE animals), pre-symptomatic (8 dpi, before clinical motor symptoms develop), onset (12 dpi, score of 1.5–2.5), peak (14 dpi, score of 3–4), and chronic (35 dpi, score of 2.5–4) (Figure 2A). Since there are no neuronal nuclei in the optic nerve, TO-PRO 3-Iodide (Topro) was used to label nuclei as an indicator of immune cell infiltration, and Iba1 to label microglia and macrophages to evaluate inflammation. In pre-symptomatic animals the optic nerve appeared normal with respect to Topro and Iba1 intensity (Figure 2B,C). By onset, hyperintense Topro clusters became visible, particularly at the optic nerve head but also with some smaller regions present along the nerve length. These clusters tended to correspond with regions of high Iba1 signal (Figure 2D,E). At peak, we observed a strong immune response in the optic nerve head, engulfing the area in Topro labelled nuclei and a strong Iba1 signal, as well as a further expansion of inflammation along the nerve (Figure 2F,G). At chronic, Topro intensity waned along the distal nerve, with a high signal still present at the optic nerve head, while the Iba1 signal remained high along the entire nerve (Figure 2H,I). Single optical sections from 3D optic nerves displayed linearly organized nuclei along the length of the optic nerve at the presymptomatic timepoint (Figure 2J). By onset, this order started to disappear, and was disrupted and the organization was completely lost at peak (Figure 2J–L). At the chronic state, ordered lines of nuclei could be observed, however, with a higher density of nuclei compared to presymptomatic animals (Figure 2M). These changes coincided with increased intensity of Iba1 immunostaining in the optic nerve (Figure 2N-Q) indicating the presence of activated microglia and macrophages.

### 2.3. Axon Degeneration in EAE Optic Nerve

**Figure 3:**
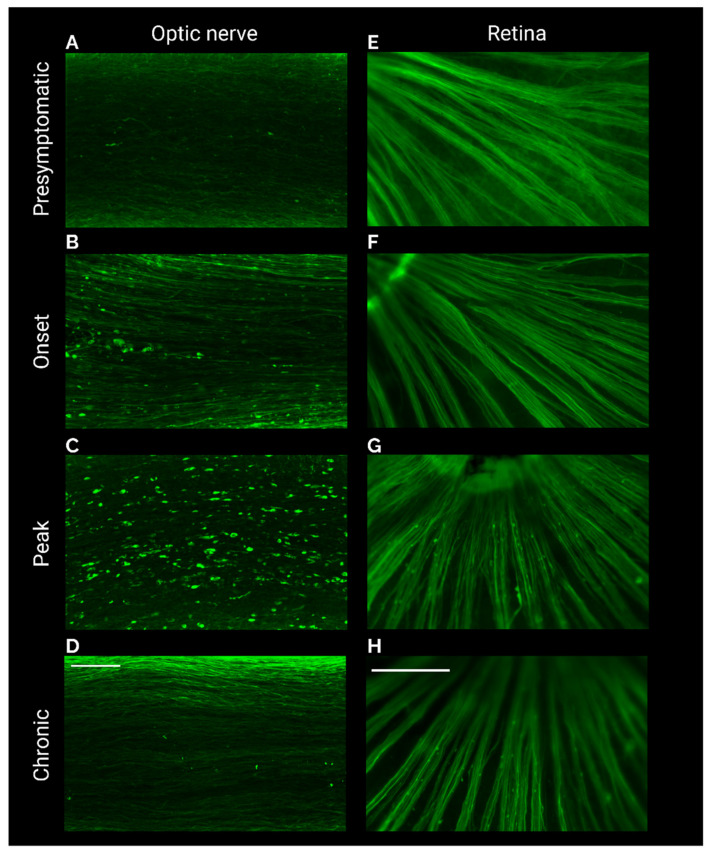
Neurofilament labelling of optic nerve retina reveals degeneration in EAE**.** (**A**,**C**,**E**,**G**) Max projections of neurofilament labelled optic nerves from presymptomatic (**A**), onset (**C**), peak (**E**), and chronic (**G**) stages of EAE. (**B**,**D**,**F**,**H**) Central retina immunostained for neurofilament from presymptomatic (**B**), onset (**D**), peak (**F**), and chronic (**H**) stages of EAE. Scale bar (**A**–**D**) = 100 μm. Scale bar (**E**–**H**) = 500 μm.

Using neurofilament antibody to label axons, optic nerve degeneration was monitored throughout the time course of EAE. Pre-symptomatic mice displayed morphologically normal axons, absent of blebs and swellings (Figure 3A). By onset, blebs could be seen along the axons in the optic nerve (Figure 3B). These blebs reached a threshold at peak but were absent at the chronic stage (Figure 3C,D). At the center of the retina where the axons project into the optic nerve head, neurofilament swellings did not appear to develop until peak, and remained present at the chronic stage (Figure 3E–H).

### 2.4. Convolutional Neural Network-Based Quantification of Axonal Blebs

**Figure 4:**
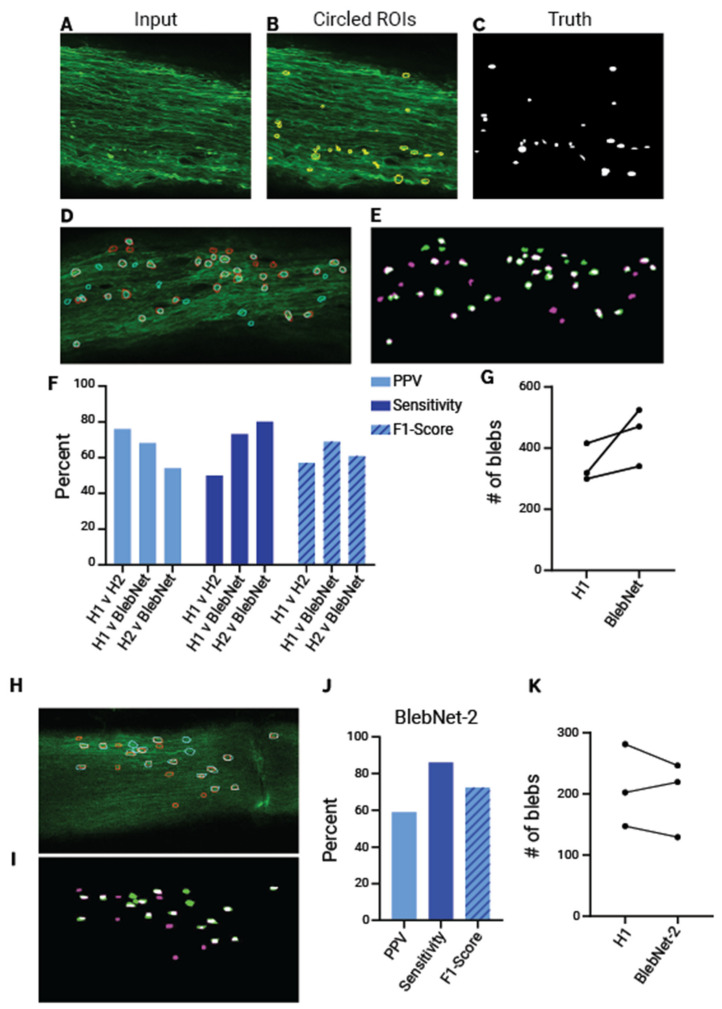
Training and validation of artificial neural net on neurofilament images. (**A**–**C**) Example of neurofilament labelled image used for training including the input image (**A**), the manually circled bleb ROIs (**B**), and the truth mask used for training based on ROIs (**C**). (**D**,**E**) Overlay of ground truth (cyan) and BlebNet identified blebs (red) over the raw input image (**D**) and as a mask where green segmentations are ground truth and magenta are BlebNet identified, and white represents the overlap (**E**). (**F**) Percentage positive predictive value, sensitivity, and F1-score for H1 vs. H1, H1 vs. BlebNet, and H2 vs. BlebNet. (**G**) Total number of blebs counted for three separate nerves between H1 and BlebNet. Overlay of ground truth (cyan) and retrained BlebNet-2 identified blebs (red) superimposed over the raw input image of neurofilament labelled nerve with optic nerve crush (**H**), and as a mask where green segmentations are ground truth, magenta are BlebNet-2 identified, and white represents the overlap (**I**). (**J**) Percentage positive predictive value, sensitivity, and F1-score for the retrained BlebNet-2 versus H1. (**K**) Total number of blebs counted for three separate nerves between H1 and the retrained BlebNet-2.

To quantify axonal blebs from neurofilament labelled optic nerves, a convolutional neural network, BlebNet, was developed, that took input images of nerves with filled ROIs around blebs as ground truth (Figure 4A–C). A UNet based architecture was employed to achieve spatial invariance in pixel segmentation, which enables improved performance in biological image segmentation tasks compared to other CNN architectures. Moreover, since the UNet has a paralleled down- and up-sampling branch, the output is a semantic segmentation that indicates the spatial location of blebs, unlike traditional CNNs which simply offer a binary classification probability output without the ability to detect where an object resides within an image [26]. Training was performed on a 2D slice-by-slice basis rather than using full 3D convolutions because full 3D convolutions require the entire optic nerve to be loaded in GPU RAM, which is computationally costly and sometimes impossible. Training slice-by-slice also allowed BlebNet to adapt to inputs of any z-stack size. To increase training speed and to save computational resources, input images were cropped into 320 × 320-pixel blocks with a 5% overlap in the x- and y-dimensions. To improve convergence, “spatial weighting” was applied to help with the segmentation of object edges. To assess the validity of BlebNet’s segmentations human and machine segmentations were visually compared (Figure 4D,E). Accuracy metrics were calculated to assess the performance of the BlebNet and the segmentation variability amongst human researchers. A second researcher (H2) was selected in addition to ground-truth (H1) to contextualize the performance of the BlebNet on positive-predictive value (PPV), sensitivity, and F1-score (an accuracy metric integrating PPV and sensitivity) over eight 320 × 320 input images. There exists inherent variability among human researchers in this optic nerve segmentation task, as the F1-score between “H1 v H2” is lower than that of “H1 v BlebNet” and “H2 v BlebNet” (Figure 4F). Notably, the sensitivity of the BlebNet was much higher than both researchers and it generally counted more total blebs than H1 suggesting higher sensitivity for feature detection (Figure 4F,G). The overall profile indicates that BlebNet performs within a similar range to human researchers (F1-score) but has higher sensitivity towards bleb identification. To ensure the tool could be used on separate but similar image sets, BlebNet was retrained on neurofilament images from optic nerve crush, a model of CNS injury where axons undergo Wallerian degeneration with axonal swelling. These nerves were imaged a year after being prepared, to assess the capacity of the tool to function on images with more noise and reduced fluorescence. Because there were fewer images in this training set, additional training images were generated through a data augmentation step that alters pre-existing images to produce similar but new images for BlebNet to quantify. Nevertheless, visual comparison of BlebNet’s segmentations and the H1 circled ROIs suggest the retrained BlebNet was able to accurately identify blebs in the new dataset (Figure 4H,I). It also achieved a high sensitivity and similar F1 score to the original version (Figure 4J). Total bleb counts for each nerve were close to the counts recorded by H1, which in context of the other metrics is suggestive of good performance after retraining (Figure 4K).

## 3. Discussion

Here, we develop a toolkit for monitoring axonal degeneration and inflammation within the optic nerve in 3D combined with an artificial neural network, BlebNet, to quantify axonal defects accurately and rapidly. Using an iDISCO protocol optimized for optic nerve, we validated various antibodies to characterize ON pathology throughout EAE. Topro labelling demonstrated extensive immune cell infiltration and disorganization of nuclei [27,28]. We report a bias towards inflammation and immune cell infiltration at the ON head, in line with previous reports demonstrating blood-brain-barrier breakdown and infiltration at the ON head in rats and guinea pigs [29,30,31]. In early stages of EAE, nuclei and Iba1 positive regions tended to overlap. However, at chronic there was sustained increase in Iba1 positivity along the entire optic nerve but the focal nuclear densities along the optic nerve length had diminished. There were also extensive axonal defects visualized as neurofilament positive blebs in the optic nerve during EAE that diminished at chronic stages, potentially reflecting the neuroaxonal loss reported in later stages of EAE [32]. While in the optic nerve these swellings are present at onset, in the retina near the optic nerve head, the swellings do not present until peak, in line with previous reports suggesting retrograde degeneration driving retinal pathology [17].

Given that manual quantification of axonal defects from using sections is a time-consuming process subject to spatial and sectioning bias, we developed BlebNet to automate this process in optical sections representing the whole 3D cleared tissue. We chose to use UNet-CNN architecture for BlebNet. UNet was previously used for a similar image segmentation task using fluorescence images to obtain information about the number and size of specific biological structures [33]. UNet enables semantic segmentation for the spatial location of features, which was necessary for our task. In its original publication, it was demonstrated to outperform the other main approach (a sliding window CNN) for image segmentation tasks [26]. Using this approach, we were able to capture fine features of axonal degeneration for quantification. This strategy could be expanded to various types of image inputs with similar features to be quantified requiring input of ground truth measurements to retrain the network. In fact, we were able to demonstrate the versatility of BlebNet to new image sets by retraining it on noisier images of neurofilament blebs from a different CNS injury model. The retraining was able to be conducted in just over 24 h using an affordable consumer tier graphics processor, demonstrated its superior processing speed in comparison to manual approaches and the ease of its implementation.

Collectively, these two approaches hereby highlighted a superior alternative to traditional sectioning techniques, subject to spatial sampling bias and data loss regarding the orientation of the nerve and the overall morphology of the tissue. Since many researchers use the optic nerve to evaluate the effectiveness of novel neuroprotective therapies in EAE, visualizing the optic nerve in 3D would be of further use in this endeavor as it provides more detail about immune cell infiltration, activation, and neurodegeneration of the optic nerve.

Although iDISCO is currently limited by the number of fluorophores and thus antibodies used to probe tissue sections—unlike traditional approaches, we expect major advancements in its applications in the near future. Overall, the iDISCO protocol is an easily implementable method that can be adapted to new antibodies, and which preserves tissue morphology and orientation for ease of imaging with a light sheet or confocal microscope. Combined with automated quantification by BlebNet, this toolkit provides an attractive alternative to tissue sectioning and manual identification of blebs and may be of use for researchers looking to understand in depth the morphology of the optic nerve in various neurodegenerative disorders.

## 4. Material and Methods

### 4.1. Experimental Animals

All housing and procedures were performed in accordance with animal use protocols approved by the Montreal Neurological Institute Animal Care and Use Committee, following the Canadian Council on Animal Care (CCAC) guidelines. All procedures involving EAE were approved by the Centre de Recherche du Centre Hospitalier de l’Université de Montréal Animal Care Committee following CCAC guidelines (approval number N19036APS).

### 4.2. Experimental Autoimmune Encephalomyelitis

EAE was induced in 8–12-week-old female C57BL/6 mice by subcutaneous injection of 200 μg MOG_35–55_ peptide in 100 μL emulsion of incomplete Freund’s adjuvant, supplemented with 4 mg/mL Mycobacterium tuberculosis, and 400 ng pertussis toxin injections intraperitoneally. Motor symptom severity was evaluated daily until the experiment end by assigning clinical scores ranging from 0 (Normal) to 5 (moribund), as previously reported [34]. Disease was classified as pre-symptomatic (8 days’ post-immunization: 8 dpi), onset (12 dpi), peak (14 dpi) and chronic (35 dpi) stage of EAE. Mice were perfused sequentially with ice cold PBS and PFA for tissue collection, and both eyes and optic nerves were surgically collected from each animal.

### 4.3. Optic Nerve Crush

Unilateral optic nerve crush was conducted on the left eye of 12- to 16-week-old adult C57BL/6 mice. Animals were anesthetized with isoflurane/oxygen (5% isoflurane, 2 L/min O_2_ induction; 2% isoflurane, 1 L/min O_2_ maintenance). A single subcutaneous injection of slow-release bupenorphrine was applied for analgesic while the animal was anesthetized. The animal’s head was shaven and cleaned with isopropyl alcohol followed by 2% chlorohexidine solution. A midline incision along the dorsum of the head was made and extended inferiorly to curve around the eye. An incision was made superior to the eye, and extraocular muscles were moved to the side, being careful not to disrupt vascular structures. The left optic nerve was visualized, and the meninges carefully dissected. The exposed optic nerve was crushed 0.5–1 mm from the optic disc with fine forceps (Dumont #5) for 10 s. Care was taken to avoid damaging the ophthalmic artery. An examination of the fundus was made after each surgery to verify the vascular integrity of the retina.

### 4.4. Whole Mount Optic Nerve iDISCO

Optic nerves were dissected removing the meninges and keeping the back of the eye attached. The tissue was dehydrated in increasing concentrations of methanol at room temperature, bleached with a −20 °C prechilled mixture of H_2_O_2_, DMSO, and methanol for 24 h at 4 °C, then rehydrated with decreasing concentrations of methanol at room temperature. The optic nerves were washed twice with 2% Triton in PBS (T-PBS) for 1 h, followed by incubation overnight at 37 °C with 10% normal goat serum (NGS) and 10% dimethyl sulfoxide (DMSO) in T-PBS (blocking buffer). The next day, optic nerves were switched to blocking buffer with heparin (10 µg/mL) and fluorophore conjugated antibodies (Neurofilament-H, MAB5256X Millipore Canada Ltd., Oakville, Canada; Iba1, 019-19741 FUJIFILM Wako, Osaka, Japan; TO-PRO 3-Iodide, T3605 Life Technologies, Burlington, Canada) and incubated at 37 °C, shaking, for up to 7 days. The antibody solution was removed, and optic nerves were washed three times, 1 h, with PBS, then washed 3× every 2 h with PBS, then incubated overnight in PBS. The next day, nerves were embedded in agar blocks (1%; Fisher scientific) and cleared using increasing concentrations of Tetrahydrofuran (THF 50%, 80% and 100%; Sigma-Aldrich Canada LTD, Oakville, Canada) followed by immersion in Benzyl Ether (DBE; Sigma-Aldrich Canada LTD, Oakville, Canada). Transparent blocks were imaged with a Lavision light sheet microscope (4× zoom) or a Leica SP8 confocal microscope and further processed with ImSpector Pro 328 and Imaris v9.5 softwares for stitching and 3D reconstruction, and ImageJ v.1.5 for straightening.

### 4.5. Retina Immunohistochemistry and Imaging

Flatmount retinas were removed from the eye globe and cut into petaloid quarters, washed with PBS, permeabilized with 0.5% Triton-PBS buffer twice then frozen for 15 min at −80 °C followed by room temperature thawing and sequential PBS washes. Retinas were incubated overnight at 4 °C in 0.5% Triton-PBS and 5% bovine serum albumin (staining solution) with anti-neurofilament primary antibody (Millipore Canada Ltd., Oakville, ON, Canada), washed sequentially with PBS, and incubated for 2 h at room temperature in Alexa Fluor 488, 568, or 647-conjugated secondary antibodies in staining solution (Life Technologies, Burlington, ON, Canada). These were sequentially washed with PBS, then imaged on a Leica SP8 confocal microscope.

### 4.6. Neural Network Architecture

A convolutional neural network with a UNet architecture (CNN-UNet) was implemented in Google’s open-source TensorFlow (1) for the segmentation of axonal blebs. The advantage of the U-shaped structure is that it provides spatially invariant pixel-wise segmentation, thus allowing for the identification of blebs through their fluorescent features and spatial positions (2) [26]. The down-sampling arm of the network consisted of three convolutional layers with sequentially increasing number of 5 × 5 filters in each layer (10, 20, 30 filters). Convolutions were performed with a stride of two to combine max-pooling in the same step. The up-sampling arm of the network integrated spatial information from previous layers to allow for spatially invariant object segmentation (2), and consisted of sequentially decreasing number of 5 × 5 filters in each layer (30, 20, 10 filters). The image was then convolved through a softmax layer with a 1 × 1 pixel kernel to generate a two-channel categorical output that was thresholded at a value of 0.5 to produce a binary segmentation. Overall, the structure adhered closely to the smaller UNet architecture optimized previously to help prevent overfitting during training, but the complexity of the model was reduced further by eliminating four of the deepest layers to prevent overfitting on our small training set [33].

### 4.7. Training

The first model was trained on a NVIDIA GeForce GTX 1070 graphics card with an intel i7 CPU processor and 32 GB RAM. The second model was trained on an NVIDIA GeForce RTX 2070 Super 8 GB graphics card with an AMD Ryzen 7 3700 processor and 16 GB of RAM. Cross-entropy loss and Jaccard index (JI) were used as metrics to monitor the performance of the CNN-UNet over multiple training iterations. Loss measures the magnitude of an error made by the neural network when comparing the output to human ground truth. Per-pixel categorical cross-entropy was chosen for the loss function. The JI measures the similarity between binarized output segmentation and ground truth. Thus, a decrease in loss and an increase in JI indicate successful task learning (4). The first training data set for the first model consisted of 720 non-bleb-containing images and 390 bleb positive images, which were sorted randomly into training (90%) and validation (10%) groups. After 400,000 epochs, training was stopped as loss started to increase, indicating overfitting (Supplemental Figure S1). The data set for the second model consisted of 182 bleb positive images. To compensate for the lower amount of training data, we increased the size of the data set by performing data augmentation. Flipping of images along X and Y axes, rescaling of pixel intensity, and addition of random noise, blur and bias field were all applied to 50% of the dataset. After 2300 epochs, training was stopped as loss started to increase, indicating overfitting (Supplemental Figure S2).

### 4.8. Post-Processing

Three post-processing steps were applied to improve the validity of segmentations when transferring from the 2D training method to final 3D segmentations. (1) x-slice thickness metric. The volume of each bleb identified must exist through a minimum of “x” number of slices in the 3D z-stack. This allows re-integration of the missing information about the spatial location of the blebs in the z-dimension. Here, the user-defined “x” value was set as 5 slices. (2) Travelling vector elimination. A common segmentation mistake by the CNN-UNet was in misidentifying extremely bright axonal segments as blebs. Fortunately, bright axonal segments have distinct structural properties from the circular features of axonal blebs, such that bright axonal segments seem to “shift” ascending through the z-stack as the microscope brings neighboring bright axonal segments into focus. Thus, if the centroid of an object shifted from slice-to-slice, this movement could be identified using vectors to determine the degree of shifting. Therefore, a movement threshold of 15 pixels was set to eliminate all shifts above that size, thus retaining the circular axonal blebs which do not shift. (3) Image opening and size threshold. Finally, to prevent small, speckled artifacts from being counted as axonal blebs, the binary image is eroded then dilated, then a user-defined threshold of 100 pixels is applied to eliminate small objects. Performance of the neural network was evaluated by the positive predictive value; the ratio of true positive outcomes to true positive and false positive outcomes, and the sensitivity; the ratio of true positive outcomes to true positive and false negative outcomes, and F1 score; the harmonic mean of PPV and sensitivity.

### 4.9. Code Availability

The code can be found at https://github.com/trissim/BlebNet (accessed on 30 October 2022).

## Supplementary Materials

The following supporting information can be downloaded at: https://www.mdpi.com/article/10.3390/ijms232314811/s1.
