# Supplementary material for: 3-Dimensional Immunostaining and Automated Deep-Learning Based Analysis of Nerve Degeneration
Source: Int J Mol Sci. 2022 Nov 26;23(23):14811. doi: 10.3390/ijms232314811 (PMC9739543; doi:10.3390/ijms232314811)

Supplemental Figure S1. Jaccard Index and Loss during BlebNet training

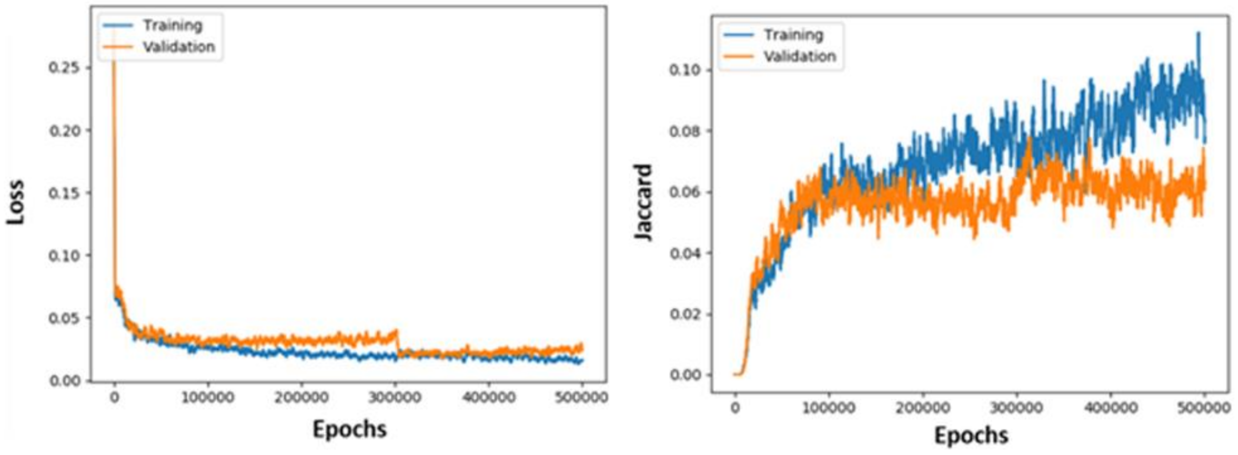

Supplemental Figure S2. Jaccard Index and Loss during BlebNet retraining

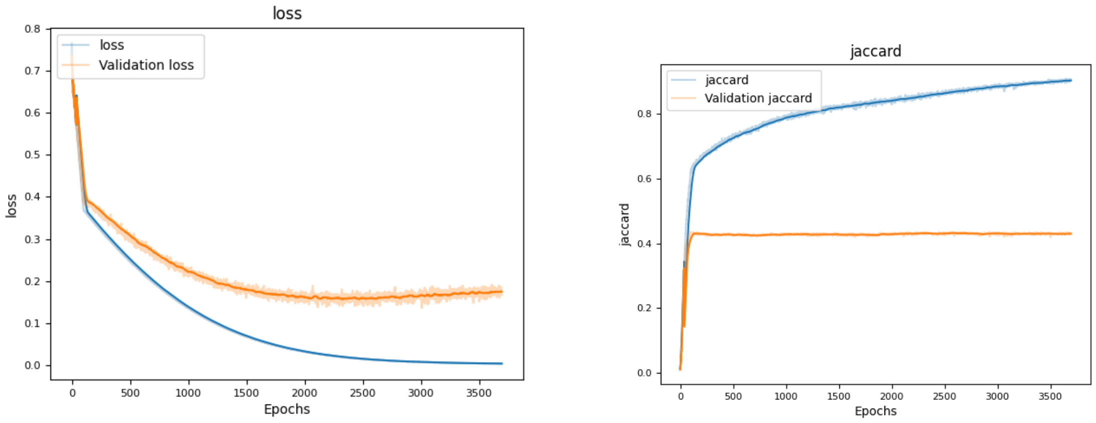

Supplement: Supplementary file 1 [file ijms-23-14811-s001.zip › ijms-1986350-supplementary.pdf]
